# Prognostic role of blood KL-6 in rheumatoid arthritis–associated interstitial lung disease

**DOI:** 10.1371/journal.pone.0229997

**Published:** 2020-03-12

**Authors:** Ho Cheol Kim, Kwang Hun Choi, Joseph Jacob, Jin Woo Song

**Affiliations:** 1 Department of Pulmonary and Critical Care Medicine, Asan Medical Center, University of Ulsan College of Medicine, Seoul, Republic of Korea; 2 Department of Respiratory Medicine, University College London, London, United Kingdom; 3 Centre for Medical Image Computing, University College London, London, United Kingdom; Nippon Medical School, JAPAN

## Abstract

Rheumatoid arthritis–associated interstitial lung disease (RA-ILD) has a variable clinical course for which predicting prognosis is difficult. However, the role of blood biomarkers in RA-ILD is ill-defined. The aim of this study was to investigate the prognostic value of Krebs von den Lungen-6 (KL-6) levels in RA-ILD patients. The medical records of 84 patients with RA-ILD were retrospectively reviewed. Plasma KL-6 levels were measured by Nanopia KL-6 assay (SEKISUI MEDICAL, Tokyo), using latex-enhanced immunoturbidimetric assay. The median follow-up period was 61 months. Mean age was 61.4 years, 45.2% were men, 44.0% were ever-smokers, and 35.7% showed a usual interstitial pneumonia (UIP) pattern on high-resolution computed tomography. The median KL-6 level at baseline was 741.2 U/mL (interquartile range, 439.7–1308.9 U/mL). On multivariate logistic regression analysis, a high KL-6 level (≥ 640 U/mL) was an independently associated with a UIP pattern (odds ratio [OR], 5.173; *P* = 0.005) with old age (OR, 1.104, *P* = 0.005). On multivariate Cox analysis, a high KL-6 level (≥ 685 U/mL) was an independent prognostic factor for the mortality (hazard ratio [HR], 2.984; *P* = 0.016) with a older age (HR, 1.061; *P* = 0.030), male sex (HR, 3.610; *P* = 0.001), lower forced vital capacity (HR, 0.957; *P* = 0.002), and a UIP pattern (HR, 4.034; *P* = 0.002). Our results suggest that high KL-6 levels might be useful as a biomarker for the presence of a UIP pattern and prognosis in patients with RA-ILD.

## Introduction

Interstitial lung disease (ILD) is a common pulmonary manifestation of rheumatoid arthritis (RA) that is associated with morbidity and mortality [1,2]. The clinical course of RA-associated interstitial lung disease (RA-ILD) is variable [3,4], and predicting prognosis is difficult. Previous studies reported that old age, male sex, reduced lung function (forced vital capacity [FVC], diffusing capacity of the lung for carbon monoxide [DLco]), a usual interstitial pneumonia (UIP) pattern, and extensive disease on high-resolution computed tomography (HRCT) are associated with poor prognosis in RA-ILD [5–9]. However, their predictive capacity may be limited by insufficient respiratory effort, complications such as emphysema or pulmonary hypertension, or interobserver variability [10,11].

Blood biomarkers are relatively easy to test independent of patient effort or reader ability. Although there are several reports on blood biomarkers in patients with idiopathic pulmonary fibrosis (IPF) [12,13], the role of biomarkers in patients with RA-ILD is ill-defined. Krebs von den Lungen-6 (KL-6) is a high-molecular-weight glycoprotein that is located in alveolar epithelial cells [14]. Since KL-6 is more highly expressed in injured or regenerating epithelial cells than in normal epithelial cells [15,16], KL-6 could be used as a biomarker of lung injury. Previous studies showed that KL-6 might be a useful biomarker in evaluation of disease severity, and prediction of prognosis in patients with IPF [17–20]. In the case of RA-ILD, Kinoshita et al. reported that KL-6 levels are correlated with the extent of the reticular opacities or total disease extent on HRCT in 47 patients with RA-ILD [21]. Lee et al. also suggested that a high KL-6 level (≥933 U/mL) might be useful as a predictor of mortality (hazard ratio [HR], 3.035; *P* = 0.023) in 62 patients with RA-UIP [22]. However, the number of patients in these studies were relatively small and the subjects were limited to the UIP group. In addition, it is not known whether KL-6 can distinguish UIP patients with a poor prognosis, amongst patients with RA-ILD [3,5,6,9]. Thus, this study aimed to investigate the role of KL-6 as a biomarker for the presence of a UIP pattern and prognosis in patients with RA-ILD.

## Materials and methods

### Study populations

From May 1995 to July 2015, 158 patients were diagnosed with RA-ILD in pulmonology department at Asan Medical Center, Seoul, Republic of Korea. Among them, 84 (biopsy-proven cases: 24) patients who had available blood sample for KL-6 measurement were included in this study. There were no differences in the baseline characteristics between patients included and those excluded from this study (S1 Table). The diagnosis of RA was made by a rheumatologist according to the revised criteria of the American College of Rheumatology [23], while that of ILD was made based on HRCT images and/or pathologic findings. Some UIP patients (n = 34, confirmed by surgical lung biopsy in 12 and by HRCT in 22) analyzed here had been included in our previous study, which used enzyme-linked immunosorbent assay (ELISA) method to measure KL-6 level [22]. The study protocol was approved by the Institutional Review Board of Asan Medical Center (approval number 2017–2280) and written informed consent for the use of blood samples for clinical research was obtained from all patients.

### Methods

Clinical and survival data for all patients were retrospectively collected from medical records, telephone interviews, and/or National Health Insurance of Korea records. All available clinical parameters were obtained within 3 months of the blood sampling time. Spirometry, diffusing capacity of the lung for carbon monoxide (DLco), and total lung capacity (TLC) were measured according to recommendations and the results are expressed as percentages of the normal predicted values [24–26]. The 6-minute walk test (6MWT) was performed according to the American Thoracic Society guidelines [27]. HRCT scans were performed per standard protocols at full inspiration without contrast enhancement. HRCT scan images were blindly reviewed by a radiologist, and the overall pattern was categorized as UIP or other. A UIP pattern was defined as a subpleural, basal predominance of reticular abnormalities, honeycombing with or without traction bronchiectasis, and the absence of findings inconsistent with a UIP pattern including extensive ground glass opacity, micronodules, discrete cysts, mosaic attenuation, or segmental/lobar consolidation [28]. Acute exacerbation (AE) was defined by the criteria of Collard et al. in 2007 [29].

### Measurement of KL-6

Blood samples were obtained by venipuncture and stored at -80°C until measurement. Plasma KL-6 levels were measured by a Nanopia KL-6 assay (SEKISUI MEDICAL, Tokyo) using a latex-enhanced immunoturbidimetric assay method as used in previous studies [30,31]. Previous study conducted in our institution reported that within-laboratory precisions of Nanopia KL-6 assay were < 2% of coefficient of variation [32].

### Statistical analysis

All values are expressed as mean ± standard deviation for continuous variables or percentages for categorical variables. Student’s t-test or the Mann-Whitney U test was used to examine continuous data, while the chi-square test or Fisher’s exact test was used to examine categorical data. The receiver operating characteristic (ROC) curve analysis was performed to confirm the optimal cut-off value of blood biomarkers for predicting a UIP pattern or survival. Logistic regression analysis and Cox proportional hazard analysis using backward elimination were used to identify independent risk factors for UIP pattern and mortality. Variables with *P* values < 0.2 on univariate analysis were entered into the multivariate models. Survival was evaluated from blood sampling date using Kaplan-Meier survival analysis and the log-rank test. Correlation analyses using Spearman’s rank correlation coefficients were performed to evaluate the relationship between KL-6 levels and lung function or exercise capacity. All *P* values were two-tailed, with statistical significance set at *P* < 0.05. All statistical analyses were performed using SPSS 20.0.

## Results

### Baseline characteristics of the study population

At the time of blood sampling, all RA-ILD patients were in a chronic stable status. The median follow-up period was 61 months (interquartile range [IQR], 27–98 months). Among 84 patients with RA-ILD, the mean subject age was 61.4 years, 45.2% were male, and 44.0% were ever-smokers (Table 1). The median KL-6 value of the total subjects was 741.2 U/mL (IQR, 439.7–1308.9 U/mL). A UIP pattern on HRCT was identified in 35.7% (n = 30) of the subjects. Treatment with steroids and/or immunosuppressive agents (such as azathioprine, cyclophosphamide, mycophenolate mofetil and cyclosporin) was administered to 74 patients (88.1%) according to the attending physician’s decision (Table 1). UIP group was less likely to receive steroids and/or immunosuppressive agents compared to the non-UIP group (*P* = 0.038). A surgical lung biopsy was performed in 24 patients and revealed that UIP was the most common histopathologic pattern (62.5%), followed by nonspecific interstitial pneumonia (NSIP) (20.8%) and organizing pneumonia (16.7%).

**Table 1:** Comparison of baseline characteristics between the UIP and the non-UIP groups of RA-ILD patients.

Characteristic	Total	UIP	Non-UIP	*P* value
Patient numbers	84	30	54	
Age, years	61.4 ± 9.4	65.8 ± 7.9	58.9 ± 9.3	0.001
Male sex	38 (45.2)	16 (53.3)	22 (40.7)	0.267
Ever-smoker	37 (44.0)	16 (53.3)	21 (38.9)	0.201
BMI, kg/m^2^	23.5 ± 3.2	23.7 ± 3.3	23.4 ± 3.2	0.616
RA duration, months	47 [9–124]	66 [30–120]	37 [7–145]	0.135
RF positivity	66 (79.5)	26 (86.7)	40 (75.5)	0.225
RF, IU/mL	125.0 [23.7–540.0]	286.0 [86.4–876.3]	83.8 [20.0–190.5]	0.015
Anti-CCP positivity	59 (79.7)	22 (88.0)	37 (75.5)	0.206
C-reactive protein, mg/dL	3.1 ± 5.2	3.2 ± 5.0	3.1 ± 5.4	0.912
KL-6, U/mL	741.2 [439.7–1308.9]	813.9 [631.9–1579.2]	628.2 [401.8–1119.3]	0.046
Pulmonary function test				
FEV_1_, % predicted	80.3 ± 17.9	82.4 ± 15.0	79.2 ± 19.4	0.431
FVC, % predicted	74.0 ± 17.2	74.7 ± 14.4	73.6 ± 18.7	0.782
DLco, % predicted	61.0 ± 20.7	56.5 ± 21.2	63.5 ± 20.2	0.141
TLC, % predicted	77.2 ± 14.8	77.0 ± 12.0	77.3 ± 16.2	0.934
6MWD, meter	417.6 ± 118.5	386.5 ± 133.2	435.9 ± 106.1	0.070
6MWT, the lowest SpO_2_, %	91.9 ± 5.3	90.3 ± 5.6	92.9 ± 5.0	0.035
Treatment regimen				0.038
Steroid only	28 (33.3)	7 (23.3)	21 (38.9)	
Steroid and/or immunosuppressive agents*	46 (54.8)	16 (53.3)	30 (55.6)	
None	10 (11.9)	7 (23.3)	3 (5.6)	
Steroid dosage, mg	12.1 ± 11.9	13.8 ± 13.7	11.4 ± 11.0	0.427

Data are presented as mean ± standard deviation, median [interquartile range], or number (%) unless otherwise indicated.

UIP, usual interstitial pneumonia; RA, rheumatoid arthritis; ILD, interstitial lung disease; BMI, body mass index; RF, rheumatoid factor; CCP, cyclic citrullinated peptide; KL-6, Krebs von den Lungen-6; FEV_1_, forced expiratory volume in 1 second; FVC, forced vital capacity; DLco, diffusing capacity of the lung for carbon monoxide; TLC, total lung capacity; 6MWD, 6-minute walk test distance; 6MWT, 6-minute walk test; SpO_2_, peripheral oxygen saturation

*Immunosuppressive agents include azathioprine, cyclophosphamide, mycophenolate mofetil and cyclosporine

### Association with the UIP pattern on HRCT

The UIP group had an older age, higher levels of KL-6 and rheumatoid factor (RF), and a lower saturation during the exercise test than the non-UIP group (Table 1). The UIP group also had reduced survival compared to the non-UIP group (S2 Table).

In the ROC analysis, KL-6 and RF were significantly associated with a UIP pattern on HRCT and the optimal cut-off levels were 640 U/mL (C-index = 0.632, *P* = 0.046) and 88 IU/mL (C-index = 0.661, *P* = 0.015). In a univariate logistic regression analysis, high levels of KL-6 (≥640 U/mL) and RF (≥88 IU/mL), and older age were associated with a UIP pattern. On multivariate analysis, high KL-6 level (odds ratio [OR], 5.173; 95% CI, 1.640–16.320; *P* = 0.005) and older age (OR, 1.104; 95% CI, 1.030–1.184; *P* = 0.005) were independently associated with a UIP pattern (Table 2).

**Table 2:** Risk factors for the presence of a UIP pattern in patients with RA-ILD assessed by logistic regression analysis.

Parameter	Odds ratio	95% confidence interval	*P* value
Univariate analysis			
Age	1.098	1.035–1.165	0.002
Male sex	1.662	0.676–4.087	0.268
Ever-smoker	1.796	0.729–4.427	0.203
BMI	1.037	0.902–1.193	0.609
RF (≥88 IU/mL)	3.970	1.455–10.837	0.007
KL-6 (≥640 U/mL)	3.538	1.301–9.622	0.013
C-reactive protein	1.005	0.923–1.094	0.910
FEV_1_	1.010	0.985–1.036	0.426
FVC	1.004	0.978–1.030	0.779
DLco	0.983	0.962–1.006	0.141
TLC	0.999	0.967–1.031	0.933
6MWD	0.996	0.993–1.000	0.075
6MWT, the lowest SpO_2_	0.912	0.835–0.996	0.041
Multivariate analysis			
Age	1.104	1.030–1.184	0.005
RF (≥88 IU/mL)	2.696	0.865–8.407	0.087
KL-6 (≥640 U/mL)	5.173	1.640–16.320	0.005

UIP, usual interstitial pneumonia; RA, rheumatoid arthritis; ILD, interstitial lung disease; BMI, body mass index; RF, rheumatoid factor; KL-6, Krebs von den Lungen-6; FEV_1_, forced expiratory volume in 1 second; FVC, forced vital capacity; DLco, diffusing capacity for carbon monoxide; TLC, total lung capacity; 6MWD, 6-minute walk test distance; 6MWT, 6-minute walk test; SpO_2_, peripheral oxygen saturation

Among the covariates significant in the univariate analysis, 6MWT, the lowest SpO_2_ was not included in the multivariate analysis because of high correlation with DLco (r = 0.733, *P* < 0.001)

### Predicting survival

Thirty-three (39.3%) patients died during follow-up. Among 22 patients with identified cause of death, the most common cause of death was disease progression (54.5%; AE, 27.3% of cause of death), followed by respiratory infection (27.3%), lung cancer (13.6%), and myocardial infarction (4.5%), respectively. Non-survivors had an older age; were more likely to be men and ever-smokers; had higher KL-6 and RF levels and lower FVC and DLco values, a shorter 6MWT distance, and lower the lowest saturations during the exercise test than survivors (Table 3).

**Table 3:** Comparison of baseline characteristics between non-survivors and survivors among patients with RA-ILD.

Characteristic	Total	Non-survivors	Survivors	*P* value
Patient number	84	33	51	
Age, years	61.4 ± 9.4	65.5 ± 8.1	58.7 ± 9.3	0.001
Male sex	38 (45.2)	21 (63.6)	17 (33.3)	0.006
Ever-smoker	37 (44.0)	21 (63.6)	16 (31.4)	0.004
BMI, kg/m^2^	23.5 ± 3.2	23.4 ± 3.2	23.7 ± 3.3	0.616
RF positivity	66 (79.5)	27 (81.8)	39 (78.0)	0.673
RF, IU/mL	125.0 [23.7–540.0]	349.0 [34.4–1410.0]	86.1 [20.0–188.3]	0.016
Anti-CCP positivity	59 (79.7)	23 (85.2)	36 (76.6)	0.376
C-reactive protein, mg/dL	3.1 ± 5.2	3.9 ± 5.4	2.7 ± 5.1	0.303
KL-6, U/mL	741.2 [439.7–1308.9]	1230.7 [644.1–1659.3]	627.1 [400.9–912.7]	0.004
Pulmonary function test				
FEV_1_, % predicted	80.3 ± 17.9	77.6 ± 18.1	82.1 ± 17.7	0.261
FVC, % predicted	74.0 ± 17.2	67.6 ± 16.2	78.2 ± 16.6	0.005
DLco, % predicted	61.0 ± 20.7	50.8 ± 21.5	67.6 ± 17.3	< 0.001
TLC, % predicted	77.2 ± 14.8	74.0 ± 15.6	79.2 ± 14.1	0.133
6MWD, meter	417.6 ± 118.5	374 ± 117.7	446.1 ± 111.2	0.007
6MWT, the lowest SpO_2_, %	91.9 ± 5.3	89.1 ± 6.1	93.8 ± 3.8	0.003
UIP pattern on HRCT	30 (35.7)	20 (60.6)	10 (19.6)	< 0.001
Treatment regimen				0.802
Steroid only	28 (33.3)	11 (33.3)	17 (33.3)	
Steroid and/or immunosuppressive agents*	46 (54.8)	19 (57.6)	27 (52.9)	
None	10 (11.9)	3 (9.1)	7 (13.7)	
Steroid dosage, mg	12.1 ± 11.9	12.1 ± 11.4	12.2 ± 12.3	0.983

Data are presented as mean ± standard deviation, median [interquartile range], or number (%) unless otherwise indicated.

RA, rheumatoid arthritis; ILD, interstitial lung disease; BMI, body mass index; RF, rheumatoid factor; CCP, cyclic citrullinated peptide; KL-6, Krebs von den Lungen-6; FEV_1_, forced expiratory volume in 1 second; FVC, forced vital capacity; DLco, diffusing capacity for carbon monoxide; TLC, total lung capacity; 6MWD, 6-minute walk test distance; 6MWT, 6-minute walk test; SpO_2_, peripheral oxygen saturation; UIP, usual interstitial pneumonia; HRCT, high-resolution computed tomography

*Immunosuppressive agents include azathioprine, cyclophosphamide, mycophenolate mofetil and cyclosporin

In the ROC analysis, KL-6 and RF were significant predictors of death and the optimal cut-off levels were 685 U/mL (C-index = 0.687, *P* = 0.004) and 88 IU/mL (C-index = 0.657, *P* = 0.016). The univariate Cox proportional hazards model showed that older age, male sex, ever-smoker status, high levels of KL-6 (≥685 U/mL) and RF (≥88 IU/mL), lower FVC and DLco, shorter 6MWT distance, and a UIP pattern on HRCT were significantly related to mortality (Table 4). On multivariate analysis, a high KL-6 level was independently associated with poor prognosis (hazard ratio [HR], 2.984; 95% CI, 1.227–7.257; *P* = 0.016); In addition, older age, male sex, lower FVC, and a UIP pattern were also independently associated with poor prognosis. When radiological honeycombing (≥ 5% of total lung extent) was included in the multivariate model instead of a UIP pattern, a high KL-6 level was also an independent prognostic factor (HR 3.235, 95% CI, 1.394–7.510, p = 0.006) along with age (HR 1.096, 95% CI 1.043–1.152, p < 0.001), ever-smoking (HR 3.179, 95% CI 1.458–6.933, p = 0.004)., FVC (HR 0.969, 95% CI 0.946–0.993, p = 0.011) and radiologic honeycombing (HR 2.884, 95% CI 1.311–6.344, p = 0.008). The diagnostic performance of high levels of KL-6 (≥685 U/mL) for mortality were as follows; sensitivity, specificity, positive predictive value, and negative predictive value were 72.7%, 56.9%, 52.1%, and 76.3%, respectively.

**Table 4:** Risk factors for the mortality in patients with RA-ILD assessed by Cox proportional hazards model.

Parameter	Hazard ratio	95% confidence interval	*P* value
Univariate analysis			
Age	1.088	1.039–1.138	< 0.001
Male sex	2.937	1.438–5.996	0.003
Ever-smoker	2.837	1.392–5.779	0.004
BMI	0.933	0.823–1.057	0.276
RF (≥88 IU/mL)	2.246	1.066–4.732	0.033
KL-6 (≥685 U/mL)	2.742	1.273–5.907	0.010
C-reactive protein	1.030	0.972–1.092	0.322
FEV_1_	0.982	0.963–1.002	0.071
FVC	0.964	0.945–0.984	< 0.001
DLco	0.953	0.934–0.972	< 0.001
TLC	0.974	0.949–1.000	0.048
6MWD	0.995	0.993–0.998	0.001
6MWT, the lowest SpO_2_	0.840	0.785–0.898	< 0.001
UIP pattern	4.073	2.003–8.281	< 0.001
Treatment with steroid and/or cytotoxic agent	1.302	0.397–4.274	0.663
Multivariate analysis			
Age	1.061	1.006–1.119	0.030
Male sex	3.610	1.644–7.931	0.001
KL-6 (≥685 U/mL)	2.984	1.227–7.257	0.016
FVC	0.957	0.931–0.984	0.002
UIP pattern	4.045	1.682–9.732	0.002

RA, rheumatoid arthritis; ILD, interstitial lung disease; BMI, body mass index; RF, rheumatoid factor; KL-6, Krebs von den Lungen-6; FEV_1_, forced expiratory volume in 1 second; FVC, forced vital capacity; DLco, diffusing capacity for carbon monoxide; TLC, total lung capacity; 6MWD, 6-minute walk test distance; 6MWT, 6-minute walk test; SpO_2_, peripheral oxygen saturation; UIP, usual interstitial pneumonia

Among the covariates significant in the univariate analysis, FEV_1_ (r = 0.889, *P* < 0.001) _,_ DLco (r = 0.605, *P* < 0.001), and TLC (r = 0.749, *P* < 0.001) were not included in the multivariate analysis because of high correlation with FVC.

### Survival prediction in the UIP group and non-UIP group

In the UIP group, KL-6 was a significant predictor for death at the optimal cut-off level of 780 U/mL (C-index = 0.765, *P* = 0.020) in ROC analysis. On multivariate Cox analysis, a high KL-6 level (≥780 U/mL) was an independent prognostic factor (HR, 4.659; 95% CI, 1.258–17.261; *P* = 0.021) with lower FVC (S3 Table). However, in the non-UIP group, KL-6 level was not associated with prognosis on ROC and univariate Cox analyses. On the contrary, male sex (HR, 5.356; 95% CI, 1.498–19.146; *P* = 0.010) and lower FVC (HR, 0.938; 95% CI, 0.901–0.977; *P* = 0.002) were independent prognostic factors in the non-UIP group (S4 Table).

### Comparison of survival between low and high KL-6 groups

**Fig 1:**
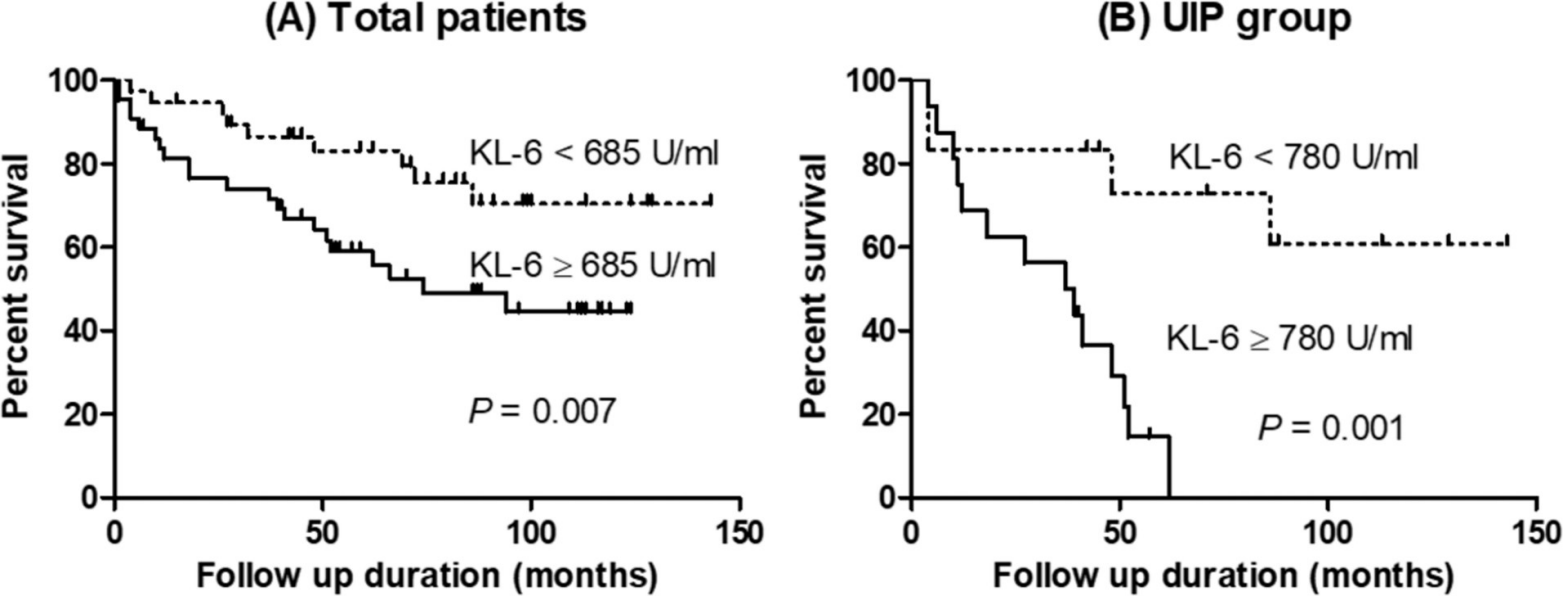
Comparison of survival curves according to KL-6 levels. (A) Total patients, (B) the UIP group. UIP, usual interstitial pneumonia.

Among the total subjects, the low KL-6 group (<685 U/mL, n = 38) had improved survival (median survival period: not reached vs. 66 months, *P* = 0.007) when compared to the high KL-6 group (≥685 U/mL, n = 46) (Fig 1A). Among the UIP group, the low KL-6 group (<780 U/mL, n = 12) also showed better prognosis (median survival period: not reached vs. 27 months, *P* = 0.001) than the high KL-6 group (≥780 U/mL, n = 18) (Fig 1B).

### Correlations between KL-6 level and lung function

**Fig 2:**
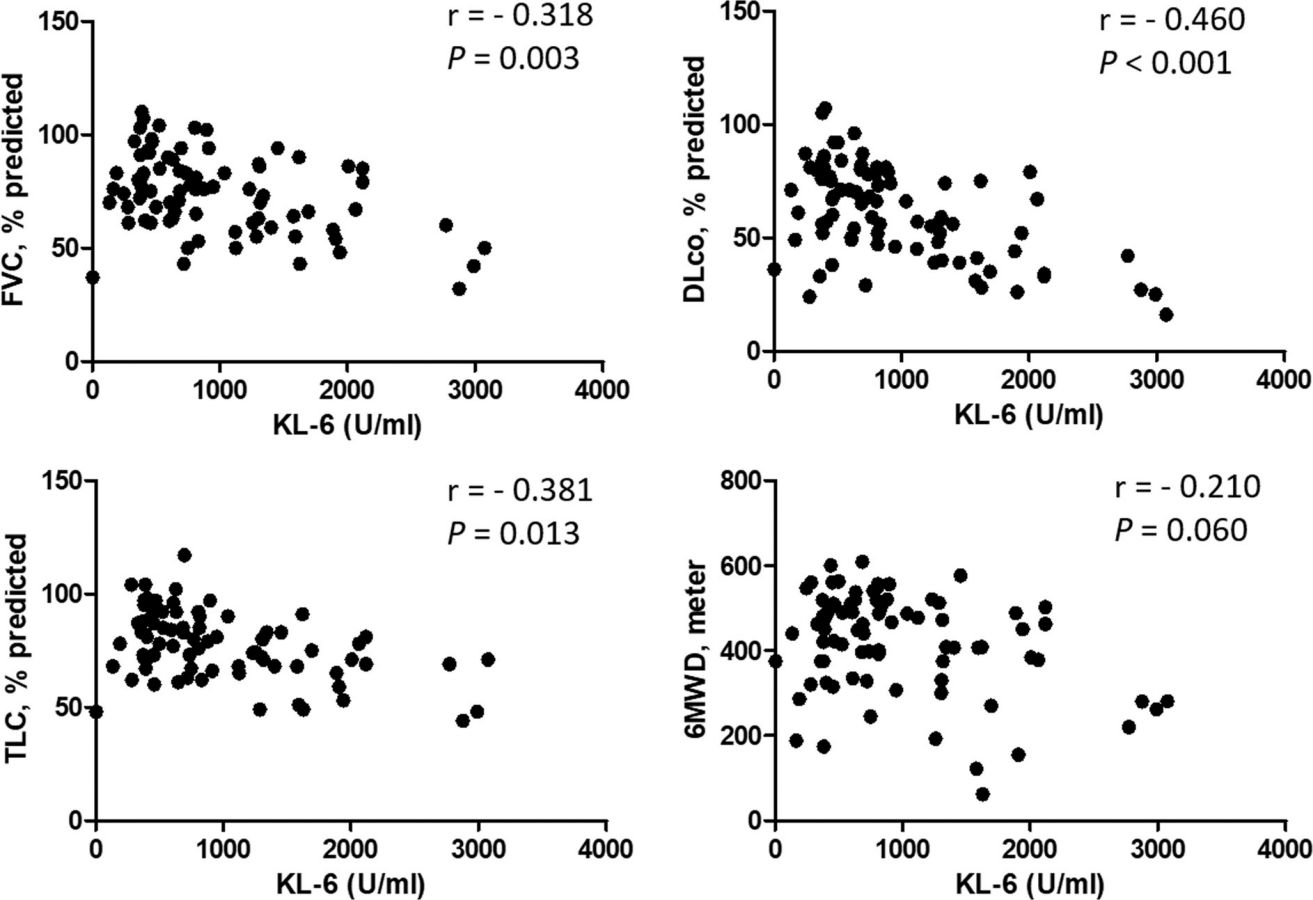
Correlation between KL-6 levels and lung function or exercise capacity. FVC, forced vital capacity; DLco, diffusing capacity for carbon monoxide; TLC, total lung capacity; 6MWD, 6-minute walk test distance.

KL-6 levels inversely correlated with FVC (r = - 0.318, *P* = 0.003), DLco (r = - 0.460, *P* < 0.001), and TLC (r = - 0.381, *P* = 0.013). KL-6 levels also showed an inverse correlation with 6MWT distance (r = -0.210, *P* = 0.060) (Fig 2). These correlation between KL-6 and lung function were maintained in both the UIP (S1 Fig) and the non-UIP groups (S2 Fig).

## Discussion

In this study, high KL-6 levels were an independently associated with a UIP pattern on HRCT in patients with RA-ILD. KL-6 levels were also inversely correlated with lung function and exercise capacity, and a high KL-6 level was an independent prognostic factor for the mortality in patients with RA-ILD, especially in the UIP group.

KL-6 is expressed on type II pneumocytes and bronchiolar epithelial cells [33]. Upon epithelial breakdown by lung injury, KL-6 is thought to leak into the blood circulation system, suggesting that it could be used as a marker of epithelial injury [16]. Several reports have shown that KL-6 would be useful for early diagnosis as well as predicting acute exacerbation, treatment outcomes, and survival in patients with IPF [17,18,34–37]. KL-6 might also serve as a diagnostic marker in connective tissue disease–associated ILD (CTD-ILD) [38]. Oguz et al., in 113 patients with CTD and 45 healthy control subjects, reported that median KL-6 values were higher in the CTD-ILD group than the CTD-without-ILD group (33.75 vs. 3.9 U/mL; *P* < 0.001) or healthy control group (33.75 vs. 3.9 U/mL; *P* < 0.008), respectively [38]. Our study showed the usefulness of KL-6 as a blood biomarker for estimating disease severity in RA-ILD. Previous results support our findings [21,31,39]. Kinoshita et al., in 47 RA-ILD patients, showed a strong positive correlation between serum KL-6 levels and the extent of reticular opacity on HRCT (r = 0.84, *P* < 0.01) [21]. Kumanovics et al., in 135 patients with CTD-ILD (104 with systemic sclerosis, 31 with myositis), also showed that KL-6 levels were negatively correlated with FVC (r = -0.32, P < 0.001) and DLco (r = - 0.55, *P* < 0.001) [39]. These results suggest that KL-6 might be useful for evaluating disease severity in cases of CTD-ILD including RA [21].

In this study, the UIP group showed reduced survival compared to the non-UIP group, and a UIP pattern on HRCT was independently associated with mortality. Our findings are consistent with previous reports [5,9]. Kelly et al., in 230 patients with RA-ILD, reported that patients with a radiologic UIP pattern had an increased all-cause mortality (relative risk, 3.9; 95% CI, 1.26–12.3; *P* < 0.018) compared to those without a UIP pattern [9]. Kim et al., in 82 patients with RA-ILD, also showed that patients with a UIP pattern on HRCT had a worse prognosis (median survival period, 3.2 vs. 6.6 years, *P* = 0.004) than those without a UIP pattern irrespective of age, sex, and lung function [5]. Therefore, it is important to differentiate the UIP group among patients with RA-ILD, but there are no known predictors of the UIP group among patients with RA-ILD. In our study, mean KL-6 level was higher in the UIP group than in the non-UIP group, and a high KL-6 level was a significant discriminating factor of a UIP pattern, suggesting that it might be useful for differentiating this group. However, Ishii et al., in 57 patients with idiopathic interstitial pneumonia (19 with UIP, 12 with NSIP) reported that serum KL-6 levels did not differ between the UIP and NSIP groups [40]. A small number of subjects and differences in the ILD disease group may have led to divergent results from our study. In addition, our current study showed that radiological honeycombing was also a poor prognostic factor, which is comparable to the prior studies [41,42].

Our results suggest that high KL-6 might be useful prognostic marker in patients with RA-ILD. Also, Previous studies suggested the usefulness of KL-6 as a prognostic biomarker in patients with UIP [22,43]. Satoh et al., in 219 patients with ILD (IIP in 152 and CTD-ILD in 67), including 183 patients (83.5%) with a UIP pattern on HRCT, showed that an elevated KL-6 level (≥1000 U/mL) was associated with a poor prognosis (HR, 2.95; *P* < 0.001) after the adjustment for age and sex [43]. Lee et al. also reported that, among 62 RA-UIP patients, those with a high KL-6 level (≥933 U/mL) had a reduced survival (median survival period: 51 vs. 96 months; *P* = 0.019) than those without a high KL-6 level irrespective of age, sex, and baseline lung function [22]. However, in our study, KL-6 was not a significant prognostic factor in the non-UIP group. The reason for this result is unclear, and one possible explanation is that the alveolar epithelium may be less damaged in the non-UIP group than in the UIP group. Repetitive microinjuries to the alveolar epithelium have been implicated in the pathogenetic cascade in idiopathic UIP (IPF) [44,45]. In a previous study, the serum levels of surfactant protein A, another biomarker of epithelial injury, were higher in the IPF group (n = 19) than in the idiopathic NSIP group (n = 12) (mean value: 104.9 vs. 46.6 ng/mL; *P* < 0.0001) [40].

This study has some limitations. First, it had a retrospective design and was conducted in a single tertiary referral center. However, the demographic features and lung function of our patients were comparable to those in other studies [5,8]. Second, a surgical lung biopsy was performed in only 24 patients (28.6% of the total subjects). However, the radiologic classification of a UIP pattern shows high agreement with the UIP pattern on the lung biopsy in patients with RA-ILD [28]. If patients with a histopathologic UIP pattern were included in the radiologic non-UIP group, they might have reduced the intergroup prognostic differences; however, the UIP group on HRCT still showed significant prognostic differences compared to the non-UIP group. Surgical lung biopsy is not usually performed in patients with CTD-ILD because histopathologic subtypes do not show treatment differences [46,47]. Third, some patients analyzed here had been included in our previous study [22]. However, in this study, we included the non-UIP group and used a different method for KL-6 measurement (latex-enhanced immunoturbidimetric assay) from the previous study (the enzyme-linked immunosorbent assay). Finally, RA activity and detailed treatment information such as type, dose, timing, and duration of medication were not considered in the analysis of prognostic factors; however, there are no proven treatments for RA-ILD, and treatment with steroids and/or cytotoxic agents were not associated with prognosis in this study.

## Conclusion

In conclusion, our results suggest that high KL-6 levels might be useful as a biomarker for the presence of a UIP pattern and prognosis in patients with RA-ILD. These findings warrant validation in further larger-scale studies.

## Supporting information

S1 Table Comparison of baseline characteristics between patients included and those excluded in the study. (DOCX)

S2 Table Comparison of treatment and survival between the UIP and the non-UIP groups among RA-ILD patients. (DOCX)

S3 Table Risk factors for the mortality in patients with RA-UIP assessed by a Cox proportional hazards model. (DOCX)

S4 Table Risk factors for the mortality in patients with RA-non-UIP assessed by a Cox proportional hazards model. (DOCX)

S1 Fig Correlation between KL-6 levels and lung function or exercise capacity in the UIP group. FVC, forced vital capacity; DLco, diffusing capacity for carbon monoxide; TLC, total lung capacity; 6MWD, 6-minute walk test distance.
(TIF)

S2 Fig Correlation between KL-6 levels and lung function or exercise capacity in the non-UIP group. FVC, forced vital capacity; DLco, diffusing capacity for carbon monoxide; TLC, total lung capacity; 6MWD, 6-minute walk test distance.
(TIF)
