# Supplementary material for: Prognostic role of blood KL-6 in rheumatoid arthritis–associated interstitial lung disease
Source: PLoS One. 2020 Mar 12;15(3):e0229997. doi: 10.1371/journal.pone.0229997 (PMC7067443; doi:10.1371/journal.pone.0229997)
Supplement: S1 Table — (DOCX) [file pone.0229997.s001.docx]

**Supporting information**

**S1 Table. Comparison of baseline characteristics between patients included and those excluded in the study**

| Characteristics^*^ | Patients included | Patients excluded | *P* value |
| --- | --- | --- | --- |
| Patient numbers | 84 | 74 |  |
| Age, years | 60.3 ± 9.2 | 61.6 ± 11.2 | 0.419 |
| Male sex | 38 (45.2) | 29 (39.2) | 0.443 |
| Ever -smokers | 37 (44.0) | 31 (41.9) | 0.785 |
| BMI, kg/m^2^ | 23.5 ± 3.2 | 23.7 ± 2.7 | 0.721 |
| RF positivity | 66 (79.5) | 54 (77.1) | 0.722 |
| RF, IU/mL | 125.0  [23.7-540.0] | 102.0  [21.0-391.0] | 0.693 |
| Anti-CCP positivity | 59 (79.7) | 52 (76.5) | 0.639 |
| C-reactive protein, mg/dL | 3.1 ± 5.2 | 1.9 ± 3.1 | 0.061 |
| Pulmonary function test |  |  |  |
| FEV_1_, % predicted | 80.0 ± 18.3 | 83.0 ± 22.0 | 0.377 |
| FVC, % predicted | 73.8 ± 17.9 | 77.1 ± 19.4 | 0.267 |
| DLco, % predicted | 61.7 ± 18.4 | 61.2 ± 20.8 | 0.892 |
| TLC, % predicted | 77.2 ± 14.8 | 77.2 ± 18.1 | 0.992 |
| 6MWD, m | 432.4 ± 115.0 | 405,1 ± 106.5 | 0.167 |
| 6MWT, the lowest SpO_2_, % | 91.6 ± 5.0 | 92.5 ± 4.9 | 0.303 |
| UIP pattern on HRCT | 30 (36.6) | 30 (41.1) | 0.565 |

Data are presented as means ± standard deviation, median [interquartile range] or number (%), unless otherwise indicated.

^*^These data are the data at that time of RA-ILD diagnosis.

BMI: body mass index, RF: rheumatoid factor, CCP: cyclic citrullinated peptide, FEV_1_: forced expiratory volume in 1 second, FVC: forced vital capacity, DLco: diffusing capacity of the lung for carbon monoxide, TLC: total lung capacity, 6MWD: six minute walk test distance, 6MWT: six minute walk test, SpO_2_: peripheral oxygen saturation, UIP: usual interstitial pneumonia, HRCT: high resolution computed tomography
