# Supplementary material for: Prognostic role of blood KL-6 in rheumatoid arthritis–associated interstitial lung disease
Source: PLoS One. 2020 Mar 12;15(3):e0229997. doi: 10.1371/journal.pone.0229997 (PMC7067443; doi:10.1371/journal.pone.0229997)
Supplement: S2 Table — (DOCX) [file pone.0229997.s002.docx]

**Supporting information**

**S2 Table. Comparison of treatment and survival between the UIP and the non-UIP groups among RA-ILD patients**

|  | Total | UIP | non-UIP | *P* value |
| --- | --- | --- | --- | --- |
| Patient numbers | 84 | 30 | 54 |  |
| Median survival period, months | Not reached | 48 | Not reached |  |
| Survival (%) |  |  |  | < 0.001 |
| 1 year | 84.5 | 70.0 | 92.6 |  |
| 3 year | 77.1 | 63.3 | 84.8 |  |
| 5 year | 57.0 | 36.8 | 84.8 |  |

Data were presented as number (%)

UIP : usual interstitial pneumonia, RA : rheumatoid arthritis, ILD : interstitial lung disease
