# Supplementary material for: Prognostic role of blood KL-6 in rheumatoid arthritis–associated interstitial lung disease
Source: PLoS One. 2020 Mar 12;15(3):e0229997. doi: 10.1371/journal.pone.0229997 (PMC7067443; doi:10.1371/journal.pone.0229997)
Supplement: S3 Table — (DOCX) [file pone.0229997.s003.docx]

**Supporting information**

**S3 Table. Risk factors for the mortality in patients with RA-UIP assessed by a Cox proportional hazards model**

| Parameters | Hazard ratio | 95% confidence interval | *P* value |
| --- | --- | --- | --- |
| Univariate analysis | | | |
| Age | 1.088 | 1.016-1.165 | 0.015 |
| Male | 1.591 | 0.648-3.902 | 0.311 |
| Ever- smokers | 1.843 | 0.732-4.636 | 0.194 |
| BMI | 0.902 | 0.762-1.067 | 0.228 |
| RF (≥ 286 IU/ml) | 1.523 | 0.619-3.747 | 0.360 |
| KL-6 (≥ 780 U/ml) | 6.681 | 1.860-23.997 | 0.004 |
| C-reactive protein | 1.005 | 0.925-1.092 | 0.905 |
| FEV_1_ | 0.978 | 0.944-1.013 | 0.221 |
| FVC | 0.942 | 0.905-0.980 | 0.004 |
| DLco | 0.959 | 0.933-0.987 | 0.004 |
| TLC | 0.952 | 0.910-0.996 | 0.033 |
| 6MWD | 0.995 | 0.992-0.999 | 0.015 |
| 6MWT, the lowest SpO_2_ | 0.924 | 0.853-1.002 | 0.056 |
| Steroid and/or cytotoxic agents | 2.174 | 0.635-7.444 | 0.216 |
| Multivariate analysis | | | |
| KL-6 (≥ 780 U/ml) | 4.659 | 1.258-17.261 | 0.021 |
| FVC | 0.955 | 0.914-0.998 | 0.041 |

RA: rheumatoid arthritis, UIP: usual interstitial pneumonia, BMI : body mass index, RF: rheumatoid factor, KL-6: Krebs von den Lungen-6, FEV_1_: forced expiratory volume in 1 second, FVC: forced vital capacity, DLco: diffusing capacity for carbon monoxide, TLC: total lung capacity, 6MWD: six minute walk test distance, 6MWT: six minute walk test, SpO_2_: peripheral oxygen saturation

Among the covariates significant in the univariate analysis, TLC (r = 0.751, *P* < 0.001) was not included in the multivariate analysis because of high correlation with FVC
