# Supplementary material for: Prognostic role of blood KL-6 in rheumatoid arthritis–associated interstitial lung disease
Source: PLoS One. 2020 Mar 12;15(3):e0229997. doi: 10.1371/journal.pone.0229997 (PMC7067443; doi:10.1371/journal.pone.0229997)
Supplement: S4 Table — (DOCX) [file pone.0229997.s004.docx]

**Supporting information**

**S4 Table. Risk factors for the mortality in patients with RA-non-UIP assessed by a Cox proportional hazards model**

| Parameters | Hazard ratio | 95% confidence interval | *P* value |
| --- | --- | --- | --- |
| Univariate analysis | | | |
| Age | 1.051 | 0.983-1.124 | 0.143 |
| Male | 4.613 | 1.405-15.148 | 0.012 |
| Ever-smokers | 3.154 | 1.026-9.700 | 0.045 |
| BMI | 0.913 | 0.741-1.124 | 0.389 |
| RF (log) | 2.188 | 1.084-4.415 | 0.029 |
| KL-6 (log) | 1.074 | 0.514-2.244 | 0.849 |
| C-reactive protein | 1.052 | 0.965-1.147 | 0.253 |
| FEV_1_ | 0.966 | 0.938-0.996 | 0.024 |
| FVC | 0.948 | 0.918-0.980 | 0.001 |
| DLco | 0.942 | 0.912-0.973 | < 0.001 |
| TLC | 0.971 | 0.934-1.009 | 0.133 |
| 6MWD | 0.995 | 0.990-1.000 | 0.052 |
| 6MWT, the lowest SpO_2_ | 0.749 | 0.654-0.857 | < 0.001 |
| Steroid and/or cytotoxic agents | 21.950 | 0.000-1321581.896 | 0.582 |
| Multivariate analysis | | | |
| Male | 5.356 | 1.498-19.146 | 0.010 |
| FVC | 0.938 | 0.901-0.977 | 0.002 |

RA: rheumatoid arthritis, UIP: usual interstitial pneumonia, BMI : body mass index, RF: rheumatoid factor, KL-6: Krebs von den Lungen-6, FEV_1_: forced expiratory volume in 1 second, FVC: forced vital capacity, DLco: diffusing capacity for carbon monoxide, TLC: total lung capacity, 6MWD: six minute walk test distance, 6MWT: six minute walk test, SpO2: peripheral oxygen saturation

Among the covariates significant in the univariate analysis, FEV_1_ (r = 0.940, P < 0.001) and TLC (r = 0.751, *P* < 0.001) were not included in the multivariate analysis because of high correlation with FVC
