# Supplementary figures and images for: Prognostic role of blood KL-6 in rheumatoid arthritis–associated interstitial lung disease
Source: PLoS One. 2020 Mar 12;15(3):e0229997. doi: 10.1371/journal.pone.0229997 (PMC7067443; doi:10.1371/journal.pone.0229997)

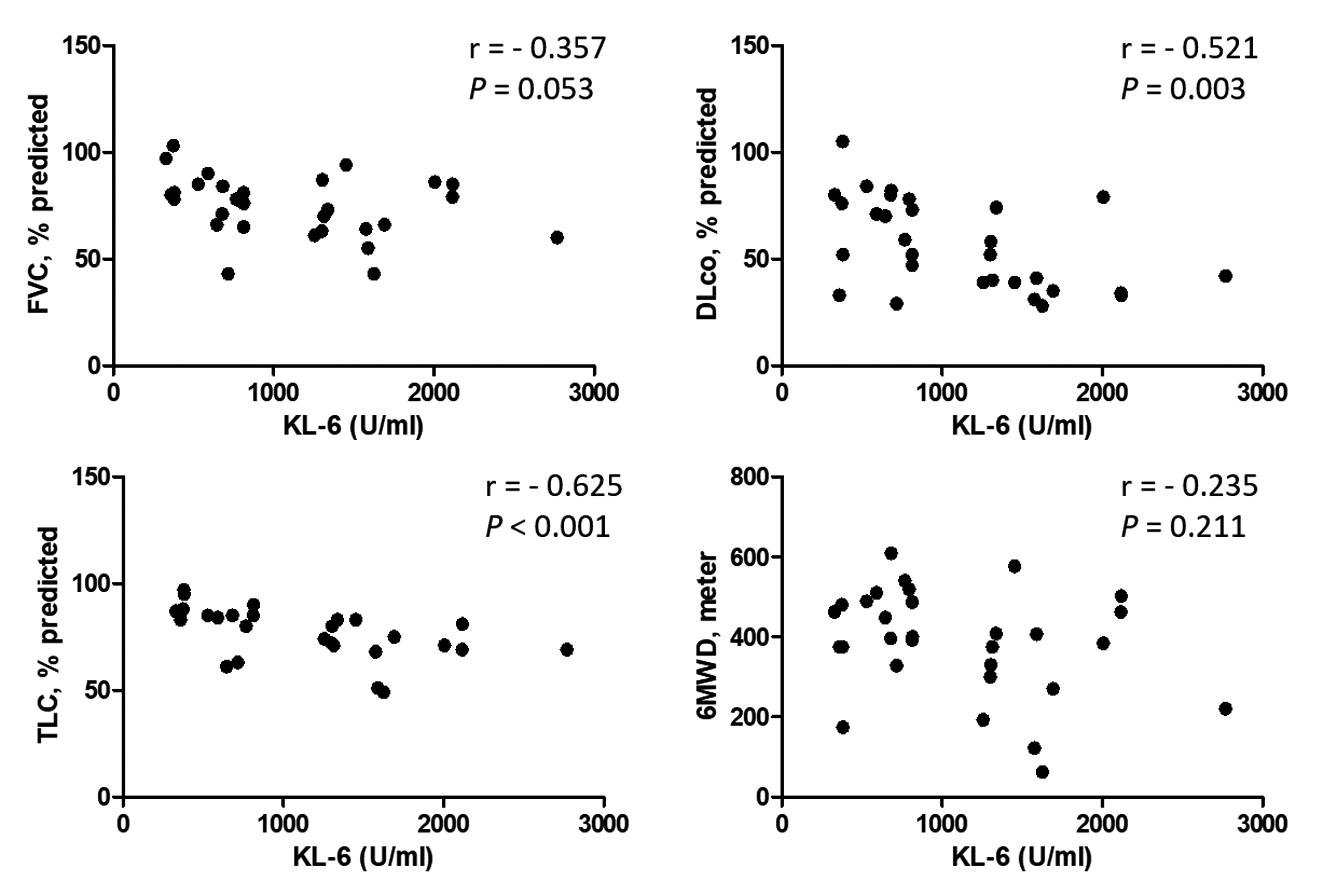

Supplement: S1 Fig — FVC, forced vital capacity; DLco, diffusing capacity for carbon monoxide; TLC, total lung capacity; 6MWD, 6-minute walk test distance. (TIF) [file pone.0229997.s005.tif]

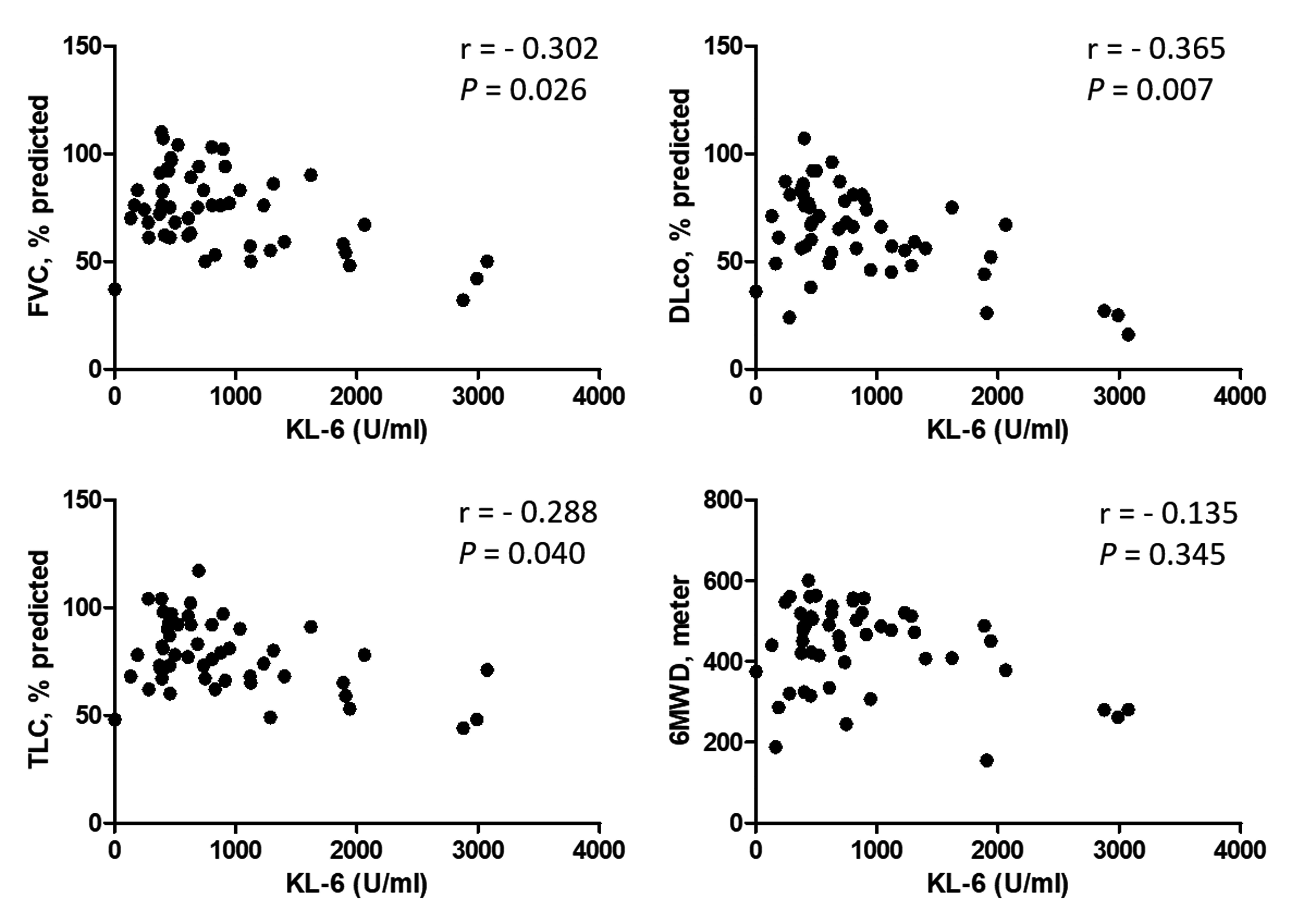

Supplement: S2 Fig — FVC, forced vital capacity; DLco, diffusing capacity for carbon monoxide; TLC, total lung capacity; 6MWD, 6-minute walk test distance. (TIF) [file pone.0229997.s006.tif]
